# A Complete Transfer Learning-Based Pipeline for Discriminating Between Select Pathogenic Yeasts from Microscopy Photographs

**DOI:** 10.3390/pathogens14050504

**Published:** 2025-05-21

**Authors:** Ryan A. Parker, Danielle S. Hannagan, Jan H. Strydom, Christopher J. Boon, Jessica Fussell, Chelbie A. Mitchell, Katie L. Moerschel, Aura G. Valter-Franco, Christopher T. Cornelison

**Affiliations:** 1 School of Data Science and Analytics, Kennesaw State University, Kennesaw, GA 30144, USA; rparke67@students.kennesaw.edu (R.A.P.); jnstrdm05@gmail.com (J.H.S.); 2 BioInnovation Laboratory, Department of Molecular and Cellular Biology, College of Science and Mathematics, Kennesaw State University, Kennesaw, GA 30144, USA; hannagand618@gmail.com (D.S.H.); cboon3@gatech.edu (C.J.B.); jfusse10@students.kennesaw.edu (J.F.); cmitch151@gmail.com (C.A.M.); katiemoerschel@gmail.com (K.L.M.); aura.valter.f@gmail.com (A.G.V.-F.)

**Keywords:** *Candida auris*, novel identification methodology, misidentification, computer vision, convolutional neural networks, microscopy, machine learning

## Abstract

Pathogenic yeasts are an increasing concern in healthcare, with species like *Candida auris* often displaying drug resistance and causing high mortality in immunocompromised patients. The need for rapid and accessible diagnostic methods for accurate yeast identification is critical, especially in resource-limited settings. This study presents a convolutional neural network (CNN)-based approach for classifying pathogenic yeast species from microscopy images. Using transfer learning, we trained the model to identify six yeast species from simple micrographs, achieving high classification accuracy (93.91% at the patch level, 99.09% at the whole image level) and low misclassification rates across species, with the best performing model. Our pipeline offers a streamlined, cost-effective diagnostic tool for yeast identification, enabling faster response times in clinical environments and reducing reliance on costly and complex molecular methods.

## 1. Introduction

Fungal pathogens pose a growing threat to public health, especially in immunocompromised individuals and healthcare settings where vulnerable patients are at heightened risk. The challenge of managing fungal infections is compounded by factors such as rising resistance to antifungal treatments, limited access to rapid and accurate diagnostic methods, and a general lack of awareness surrounding fungal pathogens [1].

One example of a particularly problematic fungal pathogen is *Candida auris*, first identified in 2009, which is now associated with severe infections across more than 35 countries [1,2,3,4]. *C. auris* exemplifies the challenges posed by pathogenic yeasts due to its multidrug resistance, and persistence in healthcare environments [5]. Similar to other pathogenic yeast species, its resilience and high mortality rates underscore the urgent need for more effective and accessible diagnostic tools [1,6,7,8].

Identifying *Candida* species accurately is a critical challenge, as several pathogenic yeasts, including *Candida auris*, *Candida glabrata*, and *Candida haemulonii*, share morphological similarities that complicate diagnosis. This leads to common misidentification between many of these species using current methods, especially when *C. auris* is involved (Table 1) [6,9]. When misdiagnoses occur, improper treatment can be prescribed, which decrease patient prognosis, as well as increase the risk of outbreaks. Current methods for distinguishing these species—such as whole-genome sequencing, biochemical assays, PCR, and MALDI-TOF MS fingerprinting—are highly accurate but often prohibitively expensive and inaccessible in many healthcare settings [4,6]. MALDI-TOF, in particular, can have costs exceeding USD 200,000, when considering setup and database access costs and even ignoring maintenance requirements. This makes this technology inaccessible to many low and middle-income regions. Alternative diagnostic methods using machine learning to classify pathogenic yeast species from microscopy images have shown promise but remain underexplored [10].

**Figure 1:**
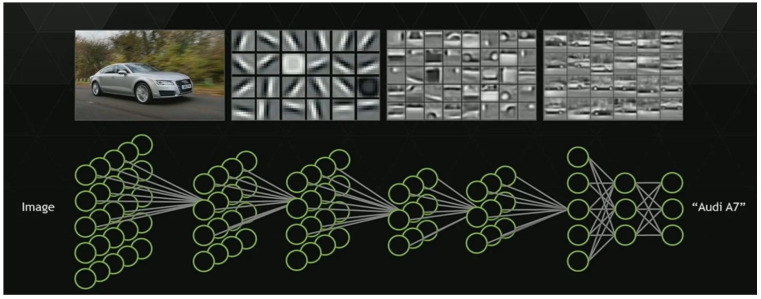
An example representation of a convolutional neural network, adapted from [14,15]. A car is used as an example, as features of a car are more intuitive than those of yeast cells would be.

Convolutional neural networks (CNN) are a popular neural network architecture in computer vision that are well known to exceed human abilities in image classification tasks. A few successful architectures include Google Net, which can classify up to 1000 image classes or CIFAR-10 which can classify up to 10 classes of tiny images with over 95% accuracy [12,13]. These networks are composed of two parts: convolutional filters and the classifier. The convolutional filters operate by passing over the pixels of an image to generate features, such as edges or sharpened regions, a process known as convolution (Figure 1) [14,15]. The classifier then takes these features as input and solves the relation between those features and the ‘class’ of the target. This involves complex mathematics, the depth of which is beyond the scope of this work. It should suffice to say that the kernel involves activation functions which impart non-linear transformation on numerical combinations of the input features to approximate the true and very non-linear polynomial function that defines the relation. The output is a set of probabilities that the instance belongs to each class, with the predicted class being the one with the highest value. For this to work, however, the model must first be trained, a process where the model is exposed to class-labeled images, from which it can learn the parameters of the best filters to use and the mathematical function to approximate the true relation. Though this is a very powerful technique, it is quite demanding, requiring significant computational resources and large quantities of high-quality images for training.

Fortunately, much of this burden can be reduced using existing convolutional filters. There is a limited set of possible image features, and sufficiently complex networks, such as those previously mentioned, can extract the majority of these. In a process called transfer learning, these extant networks are utilized by replacing the classifier but retaining the pretrained filters. This reduces training time and allows faster deployment of a model, with far less experimentation with architecture required. After the classifier has been trained relatively well, the network can be fine-tuned by allowing very small changes to the filters to be made, a process which optimizes the network to the image set and maximizes performance. Here, we seek to leverage existing convolutional neural networks but topped with a custom classifier and fine-tuned to a novel image set of micrographs, to distinguish between six related species of yeasts quickly and accurately, as a proof of concept to enable greater effort in such classification.

## 2. Materials and Methods

### 2.1. Culture Preparation

Five isolates from the CDC antibiotic resistant bank and one from the NRRL culture collection were used [16,17]. These were *Candida krusei* (AR-0397), *Candida glabrata* (AR-0319), *Saccharomyces cerevisiae* (AR-0400), *Candida haemulonii* (AR-0393), *Candida albicans* (NRRL Y-12983) and *Candida auris* (AR-0384). *Saccharomyces cerevisiae* was included to serve as a genetically and visually distinct baseline, while the others were chosen due to clinical relevance or their propensity to be confused with *C. auris* using standard methods. Stock plates using 3% Sabouraud Dextrose Agar (Thermo Fisher Scientific, Item Number: DF0109-17-1, Location: Waltham, MA, USA) of each were prepared from long-term storage stocks stored at −80 °C. Broth cultures of each species were prepared by transferring 2 to 3 colonies from stock plates into 10 mL of Sabouraud Dextrose Broth (Sigma-Aldrich, Item Number: S3306-500G, Location: Waltham, MA, USA). Cultures were incubated for 48 h at 37.0 °C with shaking, after which the cultures were stored at 3 °C for no more than three days, to minimize cell degradation.

### 2.2. Microscope Slide Preparation and Imaging

Before microscope slide preparation, each culture was concentrated by centrifuging for one minute at 3000 rpm (1693 g, as calculated). For each culture, 5 mL of supernatant was removed, and the pellet was resuspended in the tube by shaking and tapping the tube for 2–3 min. Slides were prepared with 10 µL of the concentrate, with no stains or other visualization aids. Images were taken at 40× magnification using a camera-adapted microscope (Microscope: Motic BA410E Cytology Microscope Motic Instruments Inc., Richmond, BC, Canada; Camera: Canon EOS Rebel T6i DSLR 126571, Cannon Inc., Tokyo, JapanUtility Software (Canon Inc., Tokyo, Japan) to generate an image set of 1000 images per species for further processing. While a standardized image capture protocol was followed, small amounts of variation were allowed in the quality of images, lighting and cell density, to enhance robustness of the dataset in reflecting real-world variability.

### 2.3. Image Processing and Dataset

**Figure 2:**
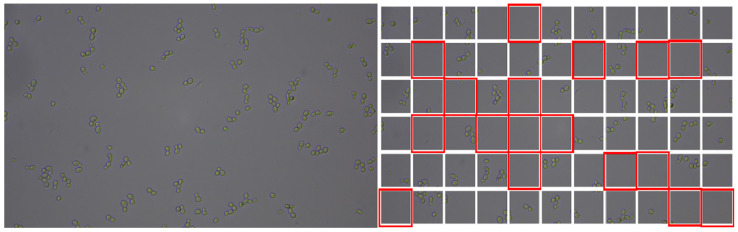
Image preprocessing step, uniformly cutting source image (*S. cerevisiae* in this example) into 66 patches, with potentially blank patches to be removed by a blank detection algorithm that used standard deviation thresholding (highlighted in red).

**Figure 3:**
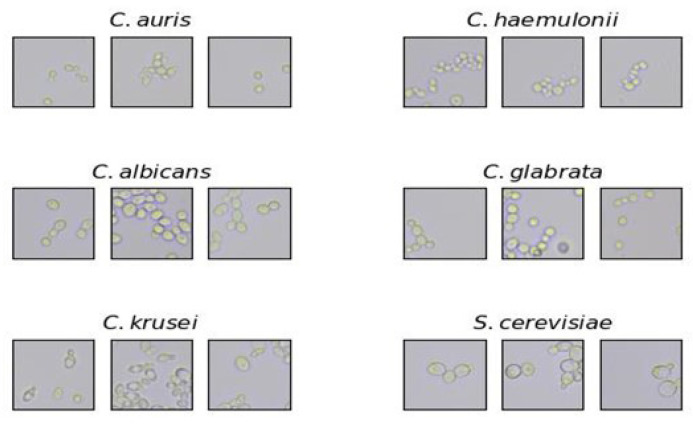
Sample patches of each species, representing some of the variation in appearance (clustering, budding, etc.) captured between patches of the same species. Note that each of these patches was correctly identified.

Each 6000 × 3368 pixel image was cut up into 512 × 512 pixel patches from the source image (Figure 2 and Figure 3) with excess discarded to meet image dimension criteria. This drastically increased the number of training samples for the machine learning algorithms. To remove potential blank patches, an elementary blank detection algorithm based on thresholding the standard deviation of pixel values was implemented. This approach allows for blank patches with low standard deviation to be dropped, based on a threshold of 2.44 standard deviations from the mean. Due to varied growth rates between species, there were varied amounts of blank images between species. To retain class balance for machine learning training, the minimal number of patches was chosen and used to construct our dataset. This resulted in a dataset with 26,585 images per *Candida* species. Of these images, 21,268 were used to train the model (the training set), 2659 were used for model tuning (the validation set) and the remaining 2658 were used to evaluate the finished model’s performance (the test set). Together, this was a train, validation, test split of about 80:10:10.

### 2.4. Model Training

**Figure 4:**
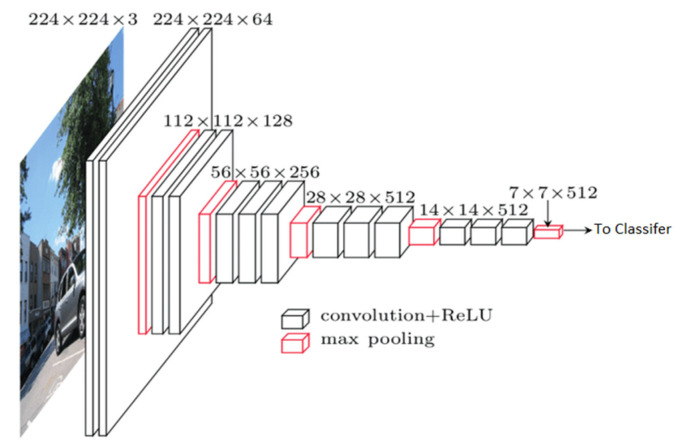
A visual representation of the VGG16 base model. Adapted from [23].

**Figure 5:**
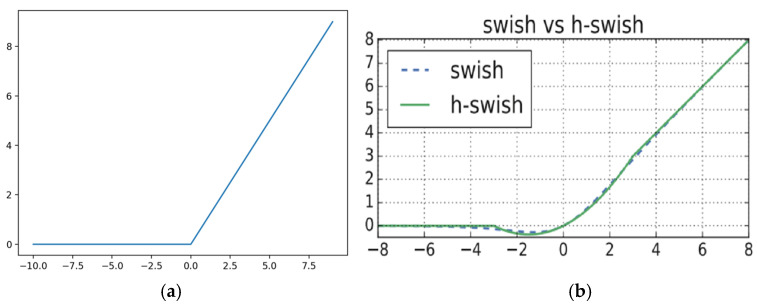
Activation functions used in neural networks. (**a**) The Rectified Linear Unit. (**b**) The non-parametric Hard Swish Function. Taken from [25,27].

Three models were trained and tested on the dataset generated. The first model was a from-scratch CNN developed using hyperband optimization to find the optimal hyperparameters [18]. Two transfer learning models, one using the VGG16 architecture as the base model, and another using the MobileNet architecture, were trained [19,20]. Both initialized the base models with the ImageNet weights [21]. Model training and fine-tuning were implemented in Python using TensorFlow (v2.12, Google LLC, Mountain View, CA, USA) and Keras (v2.12, originally developed by François Chollet, now maintained by Google LLC, Mountain View, GA, USA). For brevity’s sake, only the architecture details of the best performing model—the VGG16-based network [19,22], originally introduced by Simonyan and Zisserman, are presented (Table 2 and Table 3, Figure 4) [19,23]. For the classifier, a flatten layer was used to convert the input into a one-dimensional array for faster processing. To reduce overfitting, two dropout layers were added, one after the input layer and one following the second dense layer. These layers randomly deactivate nodes from the previous layer during training, to prevent overreliance on specific nodes that leads to overfitting. There were three dense layers (fully connected layers that work to approximate the objective function), two with 256 nodes, and one with 128 nodes. The second layer used the rectified linear unit (ReLU) as the activation function (Equation (1), Figure 5a) [22,24]. The other two used a custom-made function, a parametric form of the hard swish function (Equation (2), Figure 5b) [25,26]. The parametric function contained parameters that the model could learn, six for each node that used the function. Finally, the output layer used a SoftMax activation, which is a multinomial logistic regression that determines the final probability of each class and returns the highest as the prediction. As this was a transfer learning model, the training was performed in stages. First, the base model weights were locked and only the classifier was trained. Then, starting with the final layer of the base model, each convolutional layer was unlocked for fine-tuning, one at a time. In all of these instances, the collection of training images was randomly sorted into batches of sixteen. Each batch was passed through the model, with the weights being updated after each batch. After all the training batches were passed through, then the validation set was passed through to determine the out-of-sample loss (categorical cross-entropy, similar to mean squared error, but better suited to classification problems) and accuracy. This was repeated up to ten times (epochs), for each round of training, possibly stopping early if the loss ceased to improve. After the entirety of the model training was completed, the test set was used to evaluate the model’s final performance. A similar procedure was used to train the other two models, and the model exhibiting the highest accuracy on the test set was chosen for deployment into the final pipeline.

The Rectified Linear Unit.

(1) $$F\left(x\right)=\max\left(0,x\right)$$

The Parametric Hard Swish Function (*α_i_* are learnable parameters).

(2) $$F\left(x\right)=\alpha_{1}\left(x+\alpha_{2}\right)\min\left(\max\left(\alpha_{3}\left(x+\alpha_{4}\right),\left(x+\alpha_{5}\right)\right),\alpha_{6}\right)$$

**Figure 6:**
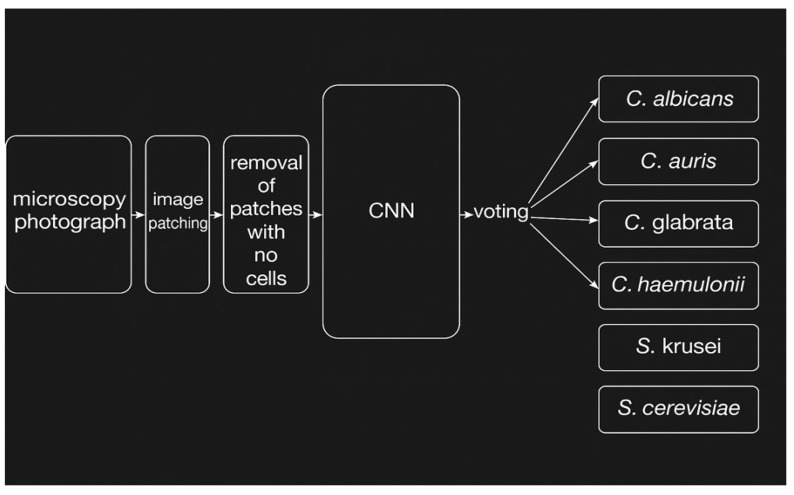
Pipeline of pre-processing and CNN model flow chart. The process begins with microscopy image acquisition, followed by patch extraction and filtering of low-content regions. Patched containing cells are passed through a convolutional neural network (CNN) for classification. Final species prediction is made via a hard-voting scheme across all patches in the image. This pipeline supports rapid, low-cost fungal diagnostics based on microscope images.

After the winning model was selected, it was integrated into a pipeline for classifying a complete micrograph (Figure 6). The pipeline begins by taking the input image and creating patches, as previously discussed. Each non-blank patch is passed through the trained model for classification. Finally, a hard voting scheme is used to classify the overall picture. Cumulatively, after the model classifies each patch, the species which received the highest probability in the greatest number of patches is selected as the prediction. To test the overall performance of the pipeline and to detect potential sources of error, the entire image library was passed through the finished pipeline.

## 3. Results

**Figure 7:**
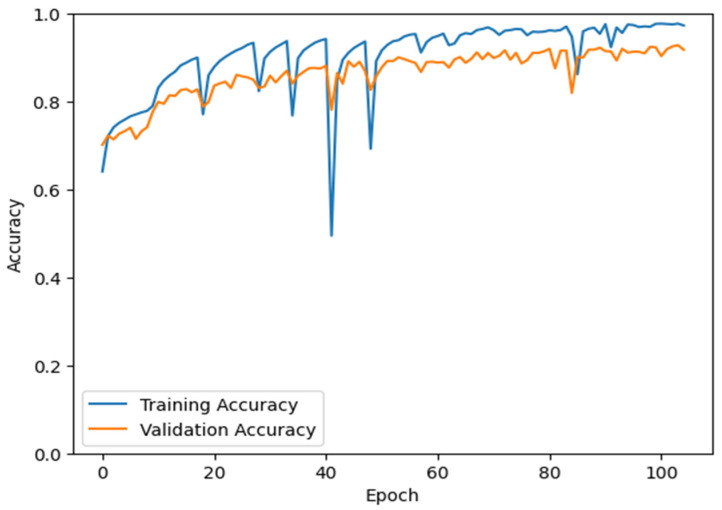
The accuracy curves of the VGG16-based model over the course of training. Each Epoch represents a complete pass over the dataset. Note that the periodic dips in accuracy occur when an additional layer of the base model is unlocked for training.

Each model achieved an overall accuracy above 70% on the image patches (Table 4), with the best performing model being the VGG16-based model, at ~93.6% accuracy. The MobileNet based model exhibited the highest range in class accuracy, showing about a 0.15 difference between its lowest, ~0.65 on *C. glabrata* to ~0.80 on its highest, *C. krusei*. The VGG16-based model presented a much tighter range of 0.035. The lowest species accuracy was *C. haemulonii* at 0.917 and the highest was *C. albicans* at 0.954. The training curves of the VGG16-based model presented as expected, with both in and out-of-sample accuracy increasing consistently throughout training (Figure 7). Note that the dips in accuracy at regular intervals are normal in the fine-tuning process. As new layers are unlocked for tuning, the combination of the additional parameters and the relatively high learning rate at the start of the training cycle causes temporary disturbances in model performance. Validation accuracy and training accuracy follow an increasing trend, with validation accuracy being slightly lower than that of the training accuracy, however being more resilient to the reductions in accuracy caused by unlocking additional layers. The confusion matrix demonstrated no signs of chronic confusion. The most prominent confusion occurred between *C. auris* and *C. haemulonii*, *C. haemulonii* and *C. glabrata*, and *S. cerevisiae* and *C. albicans* (Table 5). The assembled pipeline presented very high accuracy. Over 98% of images were classified correctly. *C. krusei* misclassified only one image. The lowest accuracy was for *C. haemulonii*, at 0.9520 (Table 2, final row). Once more, there were no signs of chronic confusion. The confusion present follows the same patterns described for the isolated model (Table 6).

## 4. Discussion

**Figure 8:**
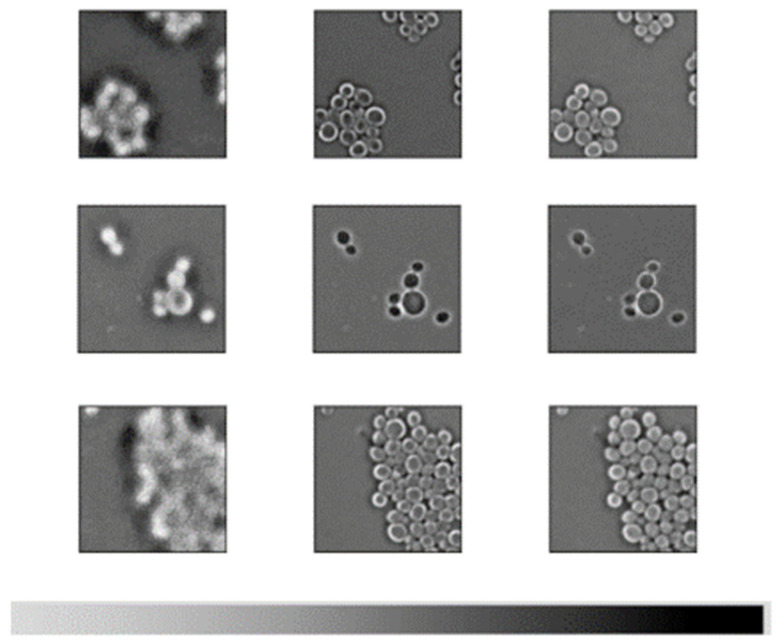
VGG16-based model feature maps which show the intensity of each channel in the RGB specification to show the detail contributed by each channel. Note that the darker a spot, the more intensely that specific pixel contributed to the learning of the model. Microscopy images acquired at 40× magnification, patches are 224 × 224 pixels.

The results of this study demonstrate that the model and pipeline successfully distinguish among multiple pathogenic yeast species from microscopy images. Of the three models tested, the VGG16-based classifier performed best, likely due to its extensive convolutional filters that capture a variety of spatial and coloration patterns in microscopic images (Figure 8). In comparison, the MobileNet-based model is optimized for mobile applications, leading to a trade-off in accuracy. The hyperband model required substantial experimentation to optimize, but ultimately did not outperform the VGG16-based approach. Deeper analysis of these two models was not performed, as the performance gap was simply too large. It should also be noted that classical statistics were not performed on the results. Big data have the benefit of avoiding prior assumptions on data distributions, instead learning them directly from the data. Additionally, the use of validation and test sets allows us to directly evaluate out of sample performance, instead of needing to rely on traditional metrics like confidence intervals and *p*-values.

**Figure 9:**
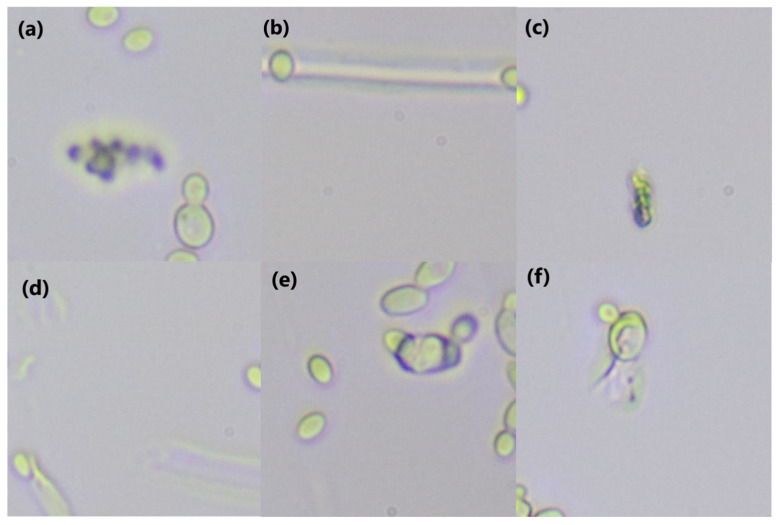
Examples of images with cell debris. Top row, left-to-right, (**a**): *C. albicans*, (**b**): *C. auris*, (**c**): *C. glabrata*. Bottom row, left-to-right, (**d**): *C. haemulonii*, (**e**): *C. krusei*, (**f**): *S. cerevisiae*. Microscopy images acquired at 40× magnification, patches are 224 × 224 pixels.

It is interesting to note that *C. haemulonii* and *C. auris* are among the most commonly misidentified species, which is not surprising, considering that this has been reported using other detection methodologies [9,10]. In general, the model does misclassify a small number of image patches. Upon further inspection, it was noted that the image quality in many of these cases was less than ideal. Cell debris or other imperfections were found in a small proportion of the images generated using the imaging methodology (Figure 9). During training, such debris would have little impact, assuming it is a relatively rare occurrence. During inference, however, such debris could pass the blank checking of the preprocessing phase. A weakness of these models is that they will issue a prediction, even if the confidence is low. Thus, given a non-blank patch with only cell debris or proliferous atypical cells, the model will give its best guess, which, in this case, is little better than a random guess. However, the hard-voting scheme protects from this when it occurs in a small number of patches. If the whole image is flawed, however, the problem persists. The simplest solution is to set a confidence threshold and either ignore the patches with low confidence or reject the entire image if the overall confidence falls below the chosen level. Another noteworthy trend amongst the misclassified images was low cell density. Each image could be split into as many as 66 patches. Images with less than 10 usable patches displayed a far higher rate of misclassification, especially when they also contained visual imperfections. This makes sense as it is a basic rule of statistics that lower precision is associated with smaller sample size. In either case, this demonstrates the importance of input image quality and standardization of the culturing, slide preparation and image capture processes.

Despite these minor and correctable or circumventable issues, these results demonstrate a proof-of-concept that a properly trained model and pipeline can, with the assistance of a camera-enabled compound microscope and a GPU-enabled computer, be used to effectively distinguish *Candida* species in a timely manner, allowing for targeted treatment to be administered rapidly. Though these organisms may not be easily distinguishable by the human eye, the machine learning models are able to pick up on subtle details that differentiate these organisms. In fact, they find underlying patterns in the image at different resolutions and consider them in an integrated way to uncover trends that can be used to discriminate between the classes. While this could have value in a clinical setting in its current form, a major improvement in this process would be enabling it to perform on micrographs prepared directly from patient skin samples, such as those from the groin, axilla, or other common colonization sites. The current requirement is that a pure culture be obtained, a process that requires at least 24–48 h. If direct patient samples can be used, the classification could be carried out in the time it takes for the preparation of the slide, capturing the image, and inferencing through the pipeline, all together in less than 30 min. It is possible that this model could perform well on such samples, but this is uncertain. Microorganisms will exhibit different morphological characteristics in different environments, and these changes may be pronounced enough to undermine the model’s predictive capabilities. If so, an obvious solution would be to retrain the model on patient sample micrograms. However, these images contain significant noise from host cells, bacteria, etc. These can prove troublesome for pure classification models.

A better choice is an object segmentation model, such as a variant Mask-RCNN [26]. This architecture consists of three different networks: a region proposal network for finding areas likely to contain cells, a classifier for identifying the type of cell, and a masking network for highlighting the pixels of the cells. The problem with this approach is that the images are typically down sampled or patched into windows. The former erases critical details and the latter leads to inaccurate masking on patch borders. Thus, we suggest modifying the approach to use a sliding window and incomplete bounding box suppression. This should allow the pipeline to detect and identify each instance from the initial image, while ignoring the background noise, all with only small increases to computational time. By integrating the current architecture into a Mask RCNN-based pipeline, this would enable the identification of the organisms in debris-ridden images, or even those of mixed cultures.

## 5. Conclusions

This study demonstrates a successful application of convolutional neural networks (CNNs) for classifying select pathogenic yeast species from microscopy images, offering a streamlined, cost-effective approach for yeast identification. The high accuracy achieved by this pipeline highlights the potential of CNN-based models to support faster, accessible diagnostic tools in clinical environments where traditional methods may be prohibitively costly or time-consuming. Considering that the cost of MALDI-TOF can exceed USD 200,000, while our camera and microscope are priced at a total of USD 4400. This makes such an option very attractive for resource-limited settings. While microscopy-based diagnostics are not currently common in clinical settings, the barrier for entry is low and many facilities use microscopy for the preliminary detection of yeasts before submitting for proper identification. The additional burden required to utilize this sort of technology would be as little as taking a photo and uploading it. With an inference time per image of about 1–2 s on a GPU-enabled system, this could be especially valuable in high-volume scenarios, such as outbreaks or triage, where immediate results are crucial to positive outcomes.

While this work provides a strong proof of concept, future efforts will aim to expand the model’s applicability by including a wider range of yeast species (such as *C. tropicalis* and *C. parapsilosis*) and isolates, as well as clade-level identification in *C. auris*, enabling more comprehensive clinical utility. Because microorganisms exhibit phenotypic plasticity in different conditions, the expanding study should include variability in factors like media type and incubation length to capture a more robust sampling of this diversity. Additionally, integrating the current classifier into an object segmentation framework, such as a Mask-RCNN variant, offers potential for isolating and identifying cells within complex or noisy samples. With modifications like a sliding window and incomplete bounding box suppression, this approach could improve identification in debris-ridden images and mixed cultures without sacrificing critical morphological details. Such advancements would further enhance the pipeline’s adaptability and robustness, supporting diverse diagnostic needs in clinical mycology.

## Figures and Tables

**Table 1:** Misdiagnosis of *Candida auris* using different identification platforms. Information current as of 27 June 2024, from CDC.gov.

Identification Platform	Diagnosis of *Candida auris*
API 20C	*Rhodotorula glutinis*
	*Candida sake*
API ID 32C	*Candida intermedia*
	*Candida sake*
	*Saccharomyces kluyveri*
BD Phoenix Yeast Identification System	*Candida haemulonii*
	*Candida catenulata*
MicroScan	*Candida famata*
	*Candida lusitaniae*
	*Candida guilliermondii*
	*Candida parapsilosis*
RapID Yeast Plus	*Candida parapsilosis*
Vitek 2 YST	*Candida haemulonii*
	*Candida duobushaemulonii*
Vitek MS MALDI-TOF (with older libraries)	*Candida lusitaniae*
	*Candida haemulonii*

Adapted from [9,11].

**Table 2:** The architecture of VGG16 base model, VGG16, used as the base model for the VGG16-based network.

Layer	Output Shape	Param #
InputLayer	(None, 224, 224, 3)	0
Conv2D	(None, 224, 224, 64)	1792
Conv2D	(None, 224, 224, 64)	36,928
MaxPooling2D	(None, 112, 112, 64)	0
Conv2D	(None, 112, 112, 128)	73,856
Conv2D	(None, 112, 112, 128)	147,584
MaxPooling2D	(None, 56, 56, 128)	0
Conv2D	(None, 56, 56, 256)	295,168
Conv2D	(None, 56, 56, 256)	590,080
Conv2D	(None, 56, 56, 256)	590,080
MaxPooling2D	(None, 28, 28, 256)	0
Conv2D	(None, 28, 28, 512)	1,180,160
Conv2D	(None, 28, 28, 512)	2,359,808
Conv2D	(None, 28, 28, 512)	2,359,808
MaxPooling2D	(None, 14, 14, 512)	0
Conv2D	(None, 14, 14, 512)	0
Conv2D	(None, 14, 14, 512)	2,359,808
Conv2D	(None, 14, 14, 512)	2,359,808
MaxPooling2D	(None, 7, 7, 512)	0

**Table 3:** The VGG16-based model architecture.

Layer	Output Shape	Param #
VGG16 Base Model	(None, 7, 7, 512)	14,714,688
Flatten	(None, 25088)	0
Dropout (50%)	(None, 25088)	0
Dense (256, PH-Swish)	(None, 256)	6,422,784
Dense (256, ReLU)	(None, 256)	65,792
Dropout (50%)	(None, 256)	0
Dense (128, PH-Swish)	(None, 128)	32,896
Output (6, Softmax)	(None, 6)	774

**Table 4:** Accuracy of our convolutional neural network models to predict the identity of select *Candida* species and *Saccharomyces cerevisiae* from the test set of micrograph image patches, as well as the complete pipeline on whole test images.

Model	*Candida* *albicans*	*Candida auris*	*Candida glabrata*	*Candida* *haemulonii*	*Candida krusei*	*Saccharomyces* *cerevisiae*	Overall
Hyperband CNN	0.8866	0.8380	0.82364	0.8442	0.9249	0.8805	0.8652
VGG16-Based CNN	0.9544	0.9200	0.9529	0.9167	0.9428	0.9482	0.9391
MobileNet-Based CNN	0.7173	0.6931	0.6572	0.7386	0.8093	0.7865	0.7337
Completed	1.0000	1.0000	1.0000	0.9914	0.9636	0.9825	0.9909
Pipeline (Whole Images)							

**Table 5:** Confusion matrix for VGG16-based model with learned weights on image patches.

Predicted Actual	*Candida* *albicans*	*Candida auris*	*Candida* *glabrata*	*Candida* *haemulonii*	*Candida krusei*	*Saccharomyces cerevisiae*
*Candida albicans*	2447	7	48	17	46	93
*Candida auris*	1	2553	15	70	17	2
*Candida glabrata*	11	51	2466	115	3	12
*Candida haemulonii*	10	138	47	2431	24	8
*Candida krusei*	11	24	2	11	2589	21
*Saccharomyces cerevisiae*	84	2	10	8	67	2487

**Table 6:** Confusion matrix for integrated pipeline on whole images.

Predicted Actual	*Candida albicans*	*Candida* *auris*	*Candida glabrata*	*Candida* *haemulonii*	*Candida krusei*	*Saccharomyces cerevisiae*
*Candida albicans*	101	0	0	0	0	1
*Candida auris*	0	69	0	0	0	0
*Candida glabrata*	0	0	42	1	0	0
*Candida haemulonii*	0	0	0	115	0	0
*Candida krusei*	0	0	0	0	53	0
*Saccharomyces cerevisiae*	0	0	0	0	2	56

## Data Availability

The data are not being made publicly available, but can be made available to individuals upon request.
